# Evaluating [^68^Ga]Ga-p14-032 as a Novel PET Tracer for Diagnosis Cerebral Amyloid Angiopathy

**DOI:** 10.3389/fneur.2021.702185

**Published:** 2021-10-27

**Authors:** Qihui Zhang, Xiaobin Zhao, Peng Lei, Hank F. Kung, Zhi Yang, Lin Zhu, Shujing Wang, Hua Zhu, Xiangxi Meng, Yunyun Duan, Li Sun, Jianwei Pan, Ruixue Ma, Haiyan Hong, Xingquan Zhao, Andrew Demchuk, Eric E. Smith, Yongjun Wang

**Affiliations:** ^1^Department of Neurology, Beijing Tiantan Hospital, Capital Medical University, Beijing, China; ^2^Department of Nuclear Medicine, Beijing Tiantan Hospital, Capital Medical University, Beijing, China; ^3^State Key Laboratory of Biotherapy, West China Hospital, Sichuan University, Chengdu, China; ^4^Department of Radiology, University of Pennsylvania, Philadelphia, PA, United States; ^5^Key Laboratory of Carcinogenesis and Translational Research, Peking University Cancer Hospital, Beijing, China; ^6^Key Laboratory of Radiopharmaceuticals, Ministry of Education, College of Chemistry, Beijing Normal University, Beijing, China; ^7^Department of Medical Imaging, Beijing Tiantan Hospital, Capital Medical University, Beijing, China; ^8^Department of Neurology, First Affiliated Hospital of Jilin University, Changchun, China; ^9^Department of Neurosurgery, The First Affiliated Hospital, Zhejiang University School of Medicine, Hangzhou, China; ^10^Department of Neurology, DongFang Hospital, Beijing University of Chinese Medicine, Beijing, China; ^11^Department of Clinical Neurosciences, University of Calgary, Calgary, AB, Canada

**Keywords:** cerebral amyloid angiopathy, amyloid-β, PET tracer, Alzheimer's disease, PET/MRI

## Abstract

**Objective:** We aimed to investigate the distribution of [^68^Ga]Ga-p14-032, a novel PET ligand that binds to vascular amyloid, in patients diagnosed clinically with probable cerebral amyloid angiopathy (CAA) compared with patients with Alzheimer's disease (AD) and normal controls (NC).

**Methods:** This longitudinal cohort study was composed of 10 subjects (three probable CAA patients, two AD patients, five NC subjects), recruited from a clinic in China. CAA patients had a history of lobar intracerebral hemorrhage (ICH) and met modified Boston criteria for probable CAA. All participants were aged at least 55 years and underwent [^68^Ga] Ga-p14-032 PET/CT or/and PET/MRI, and the Montreal Cognitive Assessment on initial assessment. Demographics were measured at baseline (diabetes, hypertension, hypercholesterolemia, ischemic stroke, and ICH). Two PET imaging experts reviewed the PET images with cortical standardized uptake value ratio (SUVr) displayed on a color scale and visually classified the images as positive or negative. The mean of SUVr was calculated using the pons as reference.

**Results:** In CAA patients, PET scans were positive in regions with higher numbers of CMBs. No significant signal was seen in AD subjects or controls. The relative [^68^Ga]Ga-p14-032 retention in the cortex was stronger in patients with CAA than AD and NC (median SUVr 2.68 ± 1.53 vs. 1.77 ± 0.08 and 0.83 ± 0.24).

**Conclusions:** Our results provide early evidence that the [^68^Ga] Ga-p14-032 PET probe binds preferentially to vascular amyloid and may be a useful tracer for diagnosing CAA.

## Introduction

Cerebral amyloid angiopathy (CAA) is a common cerebrovascular disease in the elderly (1). Because amyloid-β (Aβ) deposits in the walls of small arteries and arterioles in the cerebral cortex and leptomeninges (2), it can lead to lobar hemorrhage and cognitive decline (3, 4). The definitive diagnosis of CAA relies on biopsy or autopsy (5), but this is often not available clinically. Amyloid imaging has become an important tool in the study of CAA (6). It has been used to explore the spatial and quantitative correlation between the brain injury related to CAA and vascular amyloid (7, 8). Although current ligands can show the deposition of vascular Aβ protein, they also pass through the blood-brain barrier and bind to the Aβ protein deposited in brain parenchyma (9). Therefore, it is still difficult to differentiate CAA and Alzheimer's disease (AD) by specific binding of the tracer to Aβ protein. Although patients with CAA exhibit a relatively higher degree of posterior binding compared with overall binding than patients with AD, these differences in relative binding are not large enough to make accurate diagnoses in individual patients. What is needed are new tracers with higher specificity for vascular β amyloid.

A series of studies suggests the relationship between vascular amyloid and lobar bleeds in CAA (10–12). *In vitro* autoradiography suggests that [^68^Ga]Ga-p14-032 may be a useful PET imaging agent for selectively detecting Aβ associated with cerebral vessels in the living human brain (13). Therefore, we designed a study to address whether [^68^Ga]Ga-p14-032-PET imaging can predict sites of microbleeds in CAA patients and whether the overall [^68^Ga]Ga-p14-032 burden provides information about the currency of CAA.

## Methods

### Study Participants

We enrolled 10 subjects at least 55 years old: three probable CAA patients, two AD patients, and five normal controls (NC). The subjects were recruited from an ongoing single-center prospective registered study of evaluating [68Ga]Ga-p14-032 as a novel PET tracer for diagnosis of CAA (Beijing Tiantan Hospital, Beijing China). They underwent [^68^Ga]Ga-p14-032 PET/CT and/or PET/MRI at Beijing Cancer Hospital. Two NC subjects did not complete the PET/MRI scan because of intolerance to noise. They had brain MRI before and had no history of stroke. The three CAA subjects after symptomatic intracerebral hemorrhage (ICH) history met the criteria for probable CAA according to modified Boston criteria. The AD cases met NINCDS-ADRDA criteria. The NC subjects have no stroke or allergic history or cognitive impairment. Detailed information, including demographics, characteristics, and the Montreal Cognitive Assessment (MoCA) score were assessed as the baseline.

### Standard Protocol Approvals and Patient Consents

This study was performed with the approval of ethics committees of the participating institutions (Beijing Tiantan hospital, and Beijing Cancer Hospital) and with the informed consent of all subjects or family members.

### Imaging Acquisition and Analysis

#### Labeling

Using the freeze-drying kit containing the labeled precursor, ^68^Ga solution washed from the ^68^Ga/^68^Ga generator was used for labeling with testing of the quality of the product through pH value, radiochemical purity (thing-layer chromatography), sterility, and pyrogen. A labeled drug meeting the clinical application quality standard was used in the PET/CT or PET/MRI clinical imaging research.

#### Imaging Methods

PET/CT images of subjects were collected with the Siemens Biograph mCT flow PET/CT scanner after intravenous injection of 3–6 mCi [^68^Ga]Ga-p14-032 for 30 min and 1 h. PET images and PET/CT fusion images were obtained after reconstruction by the ordered subset expectation maximization (OSEM) algorithm. PET/MRI imaging was performed on the head with a United Imaging uPMR 790 PET/MRI scanner 2 h after the injection of [^68^Ga] Ga-p14-032. Raw image data were reconstructed using MR attenuation correction and the OSEM algorithm. The PET acquisition time was 15 min for both PET/CT and PET/MRI.

The relative retention in the cortex was expressed as SUVr. VOIs were manually delineated on the cortex and the pon of each patient. Then, the SUVmean was calculated in the voxels of each VOI. Finally, the SUVmean in the cortex was divided by the SUVmean in the pon to yield SUVr.

The hemorrhagic lesions (Hem) and the pons were manually segmented on the susceptibility weighted image (SWI) sequence for the patient. The radioactivity and volumes of these segmented areas were obtained on the registered PET image. The concentration ratio (*R*) was them obtained as


$$\begin{array}{l} R=\frac{\alpha_{Hem/V_{Hem}}}{\alpha_{pons^{/V_{pons}}}}, \end{array}$$

where α denotes the total activity of the segmented region (Bq), and *V* denotes the volume of the region.

MRI factors included diffusion-weighted imaging hyperintense lesions (DWIHLs) with associated hypointensity, or white matter hyperintensity (WMH) was evaluated visually on fluid-isointensity on apparent diffusion coefficients. White attenuated inversion recovery images were evaluated using the Fazekas scale.

The images were read and interpreted by two doctors in nuclear medicine and imaging. They were blinded to the diagnosis.

### Statistical Analysis

The statistical results were expressed in mean ± standard deviation.

## Results

### Characteristics of the Study Group

Demographic and imaging data for the 10 subjects are summarized in Table 1. As shown in the Tables 1, 2, three CAA patients and two NC subjects had microbleeds. The CAA and AD groups had a lower MoCA score (13.67 ± 5.03, 19.00 ± 2.83) than normal controls (29.20 ± 0.45). Two NC patients had incidentally discovered CMBs; one participant had two CMBs in the white matter near the border of the left lateral ventricle, and the other participant had one CMB in the left basal ganglia. The results of the two visually raters were identical.

**Table 1:** Demographics of CAA, AD, normal patients.

**Subject number**	**Cohort**	**Age**	**Sex**	**History (HTN)**	**History (ischemic stroke)**	**History (ICH)**	**MoCA score**	**[^**68**^Ga] Ga-p14-032 scan**	**SUVr**
1	NC	56	M	Y	Y	N	29	–	1.01
2	NC	69	F	Y	N	N	29	–	1.12
3	NC	57	F	N	N	N	29	–	0.56
4	NC	72	M	N	N	N	30	–	0.63
5	AD	84	M	Y	N	N	21	–	1.83
6	AD	83	F	Y	N	N	17	–	1.71
7	CAA	70	F	Y	N	Y	9	+	1.24
8	NC	68	M	Y	N	N	29	–	0.81
9	CAA	62	M	Y	N	Y	13	+	2.53
10	CAA	58	M	Y	N	Y	19	+	4.28

*HTN, hypertension; ICH, intracerebral hemorrhage; MoCA, Montreal Cognitive Assessment; NC, normal controls; AD, Alzheimer's disease; CAA, cerebral amyloid angiopathy*.

**Table 2:** Detailed neuroimaging in three CAA patients.

**Case number**	**Number of lobar CMBs (n)**	**Number of cerebellar CMBs (n)**	**Presence/distribution of cSS**	**Severity of WMH (score)**	**ICH location**
7	4	0	Y	6	Right temporal occipital lobe
9	>10	2	N	4	Left parietal lobe
10	>10	1	N	6	Right temporal lobe

*HTN, hypertension; ICH, intracerebral hemorrhage; CMBs, cerebral microbleeds; cSS, cortical superficial siderosis; WMH, matter hyperintensity*.

### Vascular Amyloid Deposition Is Detected by [^68^Ga]Ga-p14-032 Binding

**Figure 1:**
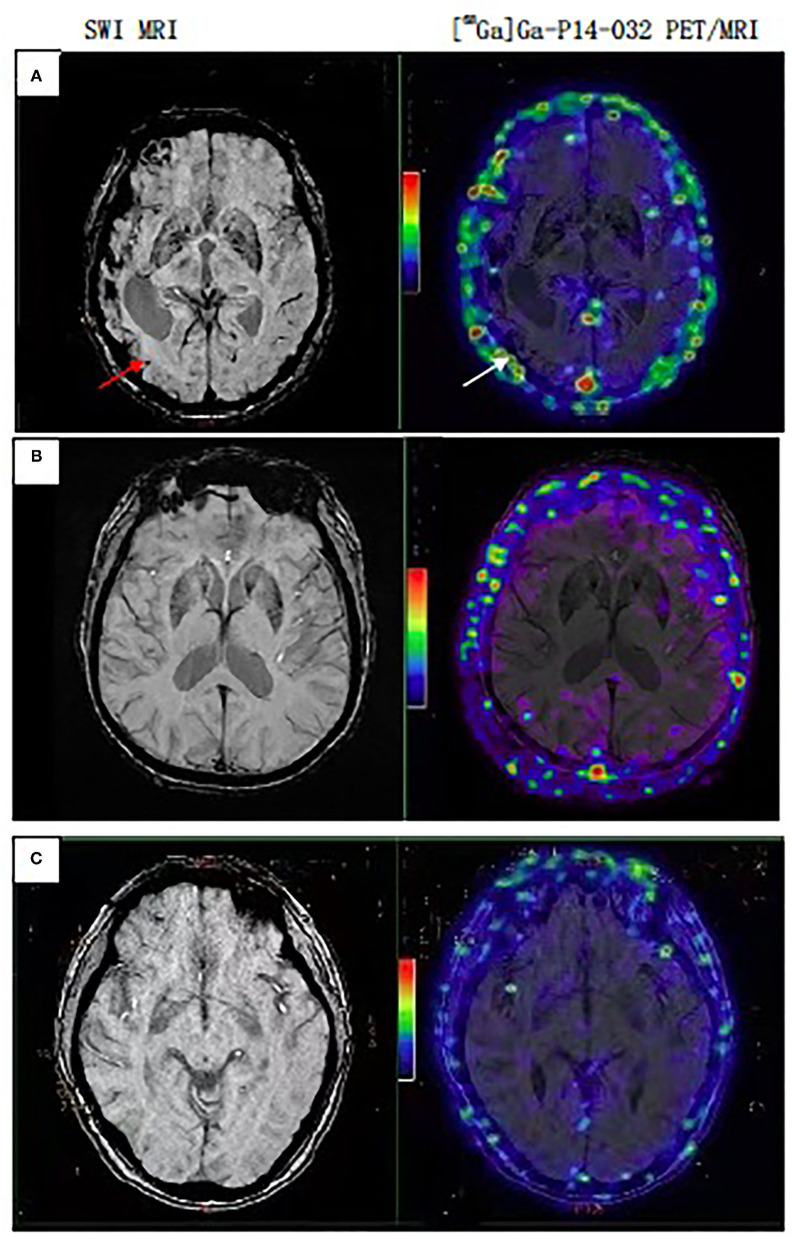
PET/MRI scan sets representative of CAA, AD, Normal subjects. **(A)** Case 7, female, 70 years, probable CAA patient, multiple CMBs on MRI, white arrow point to the same point to the same area with [^68^Ga]Ga-p14-032 retention on PET/MRI. **(B)** Case 5, male, 84 years, AD patient. **(C)** Case 3, female, 57 years, cognitive normal subject. Cases **(B,C)** show only non-specific binding in extracerebral tissues.

All AD and NC subjects were negative for [^68^Ga] Ga-p14-032 binding, and all CAA subjects were [^68^Ga] Ga-p14-032 positive by a visual read of [^68^Ga]Ga-p14-032 PET scans. In all three CAA patients, the regions with CMBs largely overlapped with regions that showed increased [^68^Ga] Ga-p14-032 uptake. It is distributed in the venous sinus, scalp, and cortex but not in the pituitary gland, white matter, brain stem, and skull. [^68^Ga]Ga-p14-032 images are shown for individual patients (Figure 1). Slices from a representative patient with probable CAA, AD, and a cognitive healthy control are shown. Global [^68^Ga]Ga-p14-032 retention was stronger in patients with CAA than those in the AD or NC groups (median SUVr 2.68 ± 1.53 vs. 1.77±0.08 and 0.08 ± 0.24) (Table 3).

**Table 3:** Description of variables from the three groups comparison.

**Variable**	**CAA (*n* = 3)**	**AD (*n* = 2)**	**NC (*n* = 5)**
Age (y)	63.33 ± 6.11	83.50 ± 0.71	64.40 ± 7.37
MoCA	13.67 ± 5.03	19.00 ± 2.83	29.20 ± 0.45
SUVr	2.68 ± 1.53	1.77 ± 0.08	0.83 ± 0.24

*SUVr, standardized uptake value ratio; MoCA, Montreal Cognitive Assessment; NC, normal controls; AD, Alzheimer's disease; CAA, cerebral amyloid angiopathy*.

## Discussion

This was the first clinical evaluation of [^68^Ga]Ga-p14-032, a novel PET ligand for vascular amyloid aggregates associated with CAA. Three CAA patients, two AD patients, and five elderly NC subjects received [^68^Ga]Ga-p14-032. We chose the pons as the reference region to calculate SUVr instead of using the cerebellum, a more widely used region in calculating amyloid PET SUVr, to avoid the confounding from cerebellar amyloid angiopathy. We found that [^68^Ga]Ga-p14-032 retention was higher in regions with CMBs in CAA patients, in which vascular amyloid deposition is expected to be the highest. Additionally, the overall retention of [^68^Ga]Ga-p14-032 in the cerebral cortex was significantly higher in patients with CAA. AD and NC subjects did not show positive regional binding of [^68^Ga]Ga-p14-032.

Case 7 had a right hemispheric dominant distribution of CMBs, and the other two CAA patients had CMBs in both hemispheres. Our preliminary study raises the possibility that [^68^Ga]Ga-p14-032 identifies vascular Aβ in areas prone to vascular rupture (14). Other studies report that CAA and associated lobar hemorrhages exhibit a preferential posterior distribution, in which vascular amyloid deposits tend to be highest (15, 16). The local accumulations of amyloid may trigger future vessel rupture and bleeding after their initial clinical presentation (17).

This study has some limitations. First, because of the sample size, the findings should be regarded as proof-of-concept. If the sample size were larger, we should be able to get more data to analysis the differences among AD and CAA *via* the conventional amyloid scan (18). Second, we did not have pathological confirmation of CAA, but used the modified Boston criteria, which have high sensitivity and specificity. Third, two of the NC subjects had incidental CMBs, which are common in older populations; however, neither had a pattern highly suggestive of CAA. If incidental CAA were present, it would tend to bias toward the null, whereas we instead found a significant difference between CAA and NC. Third, our results suggest that [^68^Ga] Ga-p14-032 is specific for vascular amyloid rather than parenchymal amyloid because retention was seen in CAA patients, but we did not have autopsy tissue with which this could be correlated. In future studies, we plan to show in the same patients that there is a differential binding of the CAA tracer vs. a non-specific beta-amyloid tracer (such as ^11^C-PiB or Florbetapir). ^11^C-PiB or Florbetapir should produce a strong signal in AD and a moderate signal in CAA, and the CAA tracer should do the opposite. Nevertheless, our findings raised the possibility that [^68^Ga] Ga-p14-032 PET, which is characteristic marker of vascular amyloid, may provide new ideas or methods for diagnosis of CAA.

## Glossary

Aβ, amyloid-β; CAA, cerebral amyloid angiopathy; SWI, susceptibility-weighted imaging; MoCA, Montreal Cognitive Assessment; PET/MRI, Positron Emission Tomography/Magnetic Resonance Imaging; AD, Alzheimer's disease.

## Data Availability Statement

The original contributions presented in the study are included in the article/Supplementary Material, further inquiries can be directed to the corresponding author/s.

## Ethics Statement

The studies involving human participants were reviewed and approved by Beijing Tiantan Hospital, Beijing Cancer Hospital. The patients/participants provided their written informed consent to participate in this study.

## Author Contributions

QZ, ES, and AD studied concept, designed, revised the manuscript for intellectual content. HK, LZ, and HH labelled the PET tracer. PL obtained the funding. ZY, SW, and HZ labelled the PET tracer and analyzed the data. XZ, XM, YD, and RM analyzed the images. XZ revised the manuscript for intellectual content. LS and JP studied coordination, contributed vital reagents, tools, and patents. YW obtained the funding and revised the manuscript for intellectual content. All authors contributed to the article and approved the submitted version.

## Funding

This study was carried out as a collaborative study supported by the Ministry of Science and Technology of the People's Republic of China (Grant Nos. 2018YFC1312300 and 2016YFC0901002). The funding source had no role in the design and conduct of the study; collection, management, analysis, and interpretation of the data; preparation, review, or approval of the manuscript; and the decision to submit the manuscript for publication.

## Conflict of Interest

The authors declare that the research was conducted in the absence of any commercial or financial relationships that could be construed as a potential conflict of interest.

## Publisher's Note

All claims expressed in this article are solely those of the authors and do not necessarily represent those of their affiliated organizations, or those of the publisher, the editors and the reviewers. Any product that may be evaluated in this article, or claim that may be made by its manufacturer, is not guaranteed or endorsed by the publisher.
