# Supplementary figures and images for: Evaluating [68Ga]Ga-p14-032 as a Novel PET Tracer for Diagnosis Cerebral Amyloid Angiopathy
Source: Front Neurol. 2021 Oct 27;12:702185. doi: 10.3389/fneur.2021.702185 (PMC8580011; doi:10.3389/fneur.2021.702185)

**Appendix**

**Supplementary Figure 1** Enlarged image of Figure 1A


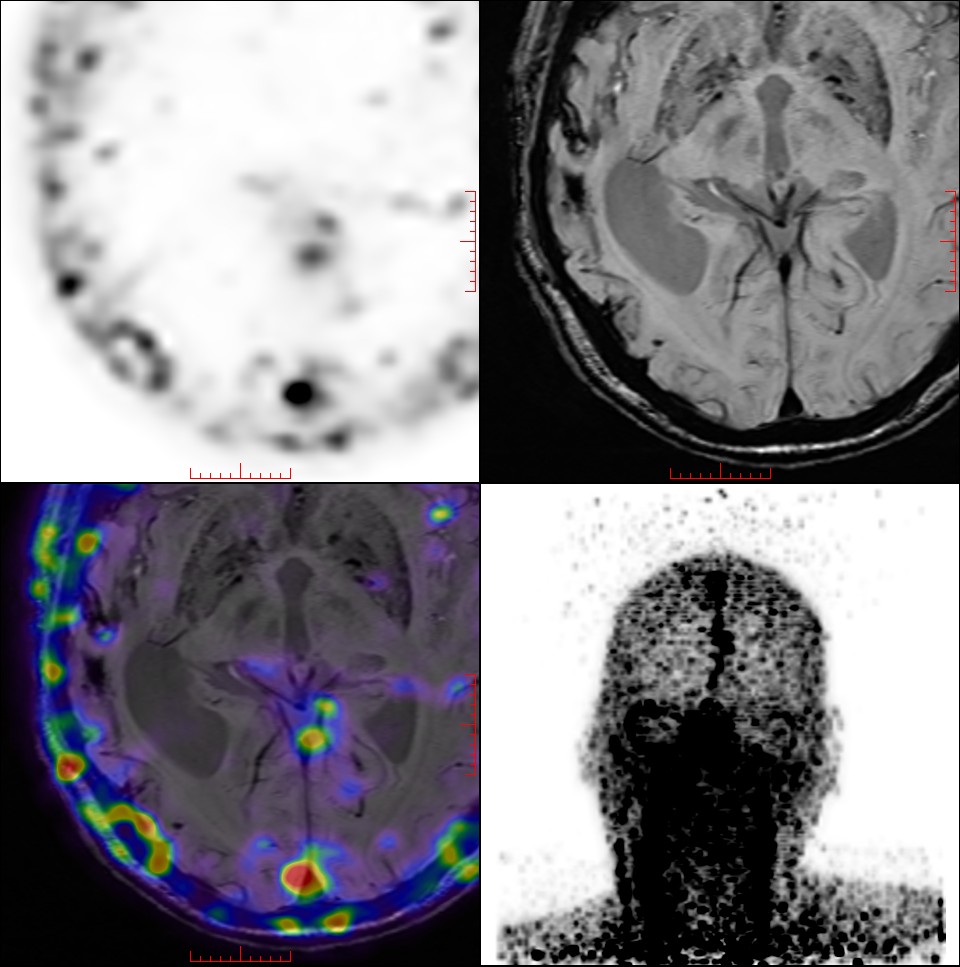

Supplement: Supplementary file 1 [file Data_Sheet_1.docx]
